# External Validation of the International Study Group for Pancreatic Surgery Complexity Grading System for Minimally Invasive Pancreatoduodenectomy: Insights From the IGOMIPS Registry

**DOI:** 10.1097/SLA.0000000000006612

**Published:** 2024-12-18

**Authors:** Niccolò Napoli, Greta Donisi, Emanuele Federico Kauffmann, Michael Ginesini, Mohammad Abu Hilal, Gian Luca Baiocchi, Umberto Bracale, Alberto Brolese, Giovanni Butturini, Roberto Coppola, Andrea Coratti, Raffaele Dalla Valle, Fabrizio Di Benedetto, Giorgio Ercolani, Giovanni Ferrari, Gianluca Garulli, Elio Jovine, Michele Mazzola, Riccardo Memeo, Carlo Molino, Luca Moraldi, Luca Morelli, Roberto Salvia, Giovanni Domenico Tebala, Vincenzo Tondolo, Roberto Ivan Troisi, Massimo Giuseppe Viola, Marco Vivarelli, Alessandro Zerbi, Ugo Boggi

**Affiliations:** * Division of General and Transplant Surgery, University of Pisa, Pisa, Italy; † Department of Biomedical Sciences, Humanitas University, Pieve Emanuele, Milan, Italy; ‡ IRCCS Humanitas Research Hospital, Rozzano, Milan, Italy; § Department of Surgery, University of Jordan, Amman, Jordan; ‖ Southampton University, Southampton, United Kingdom; ¶ Division of General Surgery, ASST Cremona, Cremona, Italy; # Department of Clinical and Experimental Sciences, University of Brescia, Brescia, Italy; ** Department of Medicine, Surgery and Dentistry, University of Salerno, Salerno, Italy; †† Department of General Surgery and Hepato-Pancreato-Biliary (HPB) Unit-APSS, Trento, Italy; ‡‡ Hepato-Biliary and Pancreatic Surgery Unit, Pederzoli Hospital, Peschiera del Garda, Verona, Italy; §§ Fondazione Campus Biomedico University, Rome, Italy; ‖‖ Department of General and Emergency Surgery and Digestive Diseases, USL Toscana Sud Est, Misericordia Hospital, School of Robotic Surgery, Grosseto, Italy; ¶¶ Hepatobiliary Surgery Unit, Department of Medicine and Surgery, University of Parma, Parma, Italy; ## Hepato-Pancreato-Biliary Surgery and Liver Transplantation Unit, University of Modena and Reggio Emilia, Modena, Italy; *** Department of Medical and Surgical Sciences, Unit of General Surgery, Morgagni-Pierantoni Hospital in Forlì, University of Bologna, Bologna, Italy; ††† Department of Oncological and Minimally Invasive Surgery, ASST Grande Ospedale Metropolitano Niguarda, Milan, Italy; ‡‡‡ Unit of General, Oncologic, Robotic, and Minimally Invasive Surgery, Infermi Hospital, Azienda Unica della Romagna, Rimini, Italy; §§§ Alma Mater Studiorum, University of Bologna and IRCCS Azienda Ospedaliero-Universitaria di Bologna, Bologna, Italy; ‖‖‖ Department of Hepato-Pancreatic-Biliary Surgery, “F. Miulli” General Regional Hospital, Acquaviva Delle Fonti, Department of Medicine and Surgery, LUM University, Casamassima, Bari, Italy; ¶¶¶ Complex Operative Unit of General and Oncologic Surgery I with High Specialization in Pancreatic Surgery, AORN A. Cardarelli, Naples, Italy; ### Division of Hepato-Biliary-Pancreatic Surgery, Azienda Ospedaliero Universitaria Careggi, Florence, Italy; **** Department of Translational Research and New Technologies in Medicine and Surgery, General Surgery Unit, University of Pisa, Pisa, Italy; †††† Unit of General and Pancreatic Surgery - Verona Pancreas Institute, Department of Engineering for Innovation Medicine (DIMI), University of Verona, Verona, Italy; ‡‡‡‡ Department of Digestive and Emergency Surgery, S. Maria Hospital Trust, Terni, Italy; §§§§ Digestive Surgery Unit, Department of Translational Medicine, Catholic University of the Sacred Heart, Rome, Italy; ‖‖‖‖ Department of Clinical Medicine and Surgery, Division of HBP, Minimally Invasive and Robotic Surgery Transplantation Center, Federico II University Hospital, Naples, Italy; ¶¶¶¶ Department of Surgery, Cardinale G. Panico Hospital,Tricase (LE), Italy; #### Division of Hepatobiliary, Pancreatic, and Transplantation Surgery, Polytechnic University of Marche, Ospedali Riuniti delle Marche, Ancona, Italy

**Keywords:** IGOMPIS, ISGPS complexity score, laparoscopic pancreatoduodenectomy, minimally invasive pancreatoduodenectomy, robotic pancreatoduodenectomy

## Abstract

**Objective:**

To validate the International Study Group for Pancreatic Surgery (ISGPS) complexity grading system for minimally invasive pancreaticoduodenectomy (MIPD).

**Background:**

Although concerns about patient safety persist, MIPD is gaining popularity. The ISGPS recently introduced a difficulty grading system to improve patient selection by aligning procedural complexity with surgeon and center expertise.

**Methods:**

Data from MIPD cases reported in the IGOMIPS registry (October 2019–February 2024) were analyzed, with severe postoperative complications as the primary outcome. Logistic regression was used to identify risk factors for complications.

**Results:**

Of the 771 MIPD cases, 426 (55.3%) were analyzed. A pancreatic duct size ≤3 mm was the only significant risk factor for severe complications (odds ratio = 2.22, *P* = 0.0001). Most cases (n = 255; 59.9%) were classified as grade C complexity, whereas 22 (5.1%) were classified as grade A. Severe postoperative complications increased with complexity (grade A, 31.8%; grade B, 36.3%; grade C, 48.6%; *P* = 0.0091). For grade A complexity, the outcomes were consistent across surgeons and centers. Grade B outcomes were similar between grade B and C centers but superior to grade A centers. In grade C cases, outcomes were comparable between grade A and B centers, with improvements at grade C centers. Grade A ISGPS experience correlated strongly with mismatches between planned and performed procedures (grade A, 15.0%; grade B, 3.0%; grade C, 3.1%; *P* < 0.0001), including total pancreatectomy (grade A, 11.5%; grade B, 1.2%; grade C, 3.1%; *P* = 0.0005).

**Conclusions:**

The ISGPS complexity grading system effectively predicted MIPD outcomes, supporting better patient selection and alignment of complexity with surgical expertise.

Pancreatoduodenectomy (PD) remains technically challenging. Despite advancements, outcomes of open PD remain suboptimal, with many patients experiencing severe postoperative complications.^1,2^ Minimally invasive PD (MIPD) has recently emerged as a viable alternative in select cases.^3,4^ However, concerns regarding patient safety and oncologic efficacy have arisen,^5,6^ prompting critical discussions on patient selection,^7^ surgeon training,^8^ and the steep learning curve associated with this procedure.^9^ In this evolving context, a standardized difficulty grading system is necessary to align patient selection with institutional experience, ensuring both safety and optimized outcomes.

Two difficulty grading systems have recently been introduced: one specific to robotic PD (PD-ROBOSCORE) and another designed for broader use in all MIPD cases,^10,11^ the latter developed by the International Study Group for Pancreatic Surgery (ISGPS). The ISGPS system classifies difficulty based on patient, surgeon, and center-related factors using an A-B-C grading framework. Patient-related factors included anatomic considerations (such as the main pancreatic and bile duct diameters), tumor characteristics (such as vascular involvement), and conditional factors [eg, body mass index (BMI) ≥30 kg/m² or prior complex upper abdominal surgery]. In this system, grade A reflects the absence of risk factors, grade B indicates the presence of one risk factor, and grade C indicates multiple risk factors or vascular involvement. Similarly, this grading applies to surgeons (A: <40 cases; B: 41–79 cases; C: ≥80 cases) and centers (A: <10 cases; B: 10–30 cases; C: >30 cases), based on overall and annual case volumes.

Based on the ISGPS score, surgeons and centers classified as level A should manage MIPD in patients with grade A complexity. Surgeons and centers at level B are suitable for handling patients with grades A and B, whereas those at level C can manage patients of any complexity grade.

Although the ISGPS grading system is based on a literature review and expert consensus, it is yet to be clinically validated. This study aimed to validate the ISGPS score using data from the Italian prospective observational registry on pancreatic surgery (IGOMIPS).^12,13^

## METHODS

The IGOMIPS registry (https://www.yoursuite.it/IGOMIPS/) is a prospective Italian database tracking operative and 90-day postoperative outcomes of minimally invasive pancreatic resections based on an intention-to-treat approach (ie, procedures are declared the day before surgery). Furthermore, it records the consecutive case numbers per surgeon and center, including cases performed before the establishment of the registry. Launched in 2019 following Ethics Committee approval at the Humanitas Institute (authorization 2167), it is registered with the Registry of Patient Registries (https://www.ahrq.gov/ropr/index.html). Additional approval was obtained from the local Ethics Committees at the participating centers.^12,13^ This study followed the Declaration of Helsinki and adhered to Strengthening the Reporting of Observational studies in Epidemiology guidelines.^14,15^

### Study Design

The IGOMIPS registry was queried for MIPD procedures performed between October 1, 2019 and February 2, 2024. Patients with complete data were included in the study. Procedures were categorized as A, B, and C based on the ISGPS classification, and outcomes were compared across surgeons and centers. The primary outcome was the incidence of severe postoperative complications (> grade 2 on the Clavien-Dindo scale).^16^ Predictors of severe postoperative complications were evaluated using a logistic regression analysis. Pancreas-specific complications were classified according to ISGPS definitions.^17–20^ Validation of the ISGPS grading system was based on the hypothesis that outcomes for grade A procedures should be similar regardless of surgeon or center experience. For grade B procedures, outcomes were expected to improve with more experienced surgeons and centers (categories B and C), whereas, for grade C procedures, category C surgeons and centers were anticipated to outperform those in categories A and B.

### Variables for International Study Group for Pancreatic Surgery Score Calculation

While the IGOMIPS registry provided more data than required for the ISGPS, 2 parameters were not prospectively recorded: common bile duct diameter and vascular contact. Consequently, patients without a prior cholecystectomy or ongoing biliary obstruction were classified as having a thin bile duct. Patients who underwent vascular resection were assumed to have vascular contacts. Additional procedural data and information on the surgeon and center experience were collected. Center volume was calculated based on the median annual volume during the study period, and the surgeon and center categories were assigned using the lower value, as per the ISGPS guidelines.

### Statistical Methods

Nominal variables were reported as frequencies and percentages, while continuous variables were expressed as mean ± SD for normally distributed data or median with interquartile range (IQR) for non-normally distributed data, as determined using the Shapiro-Wilk test.

Procedure complexity was validated by comparing intraoperative and postoperative outcomes across different difficulty levels, while center experience was validated by comparing outcomes across varying experience levels. For patient selection validation, the outcomes were stratified by procedural complexity and center experience.

Continuous variables were compared using the Wilcoxon rank-sum test for non-normally distributed data or *t* test and analysis of variance test for normally distributed data, according to the results of the skewness analysis for each variable. Welch analysis of variance was applied to detect unequal variances using the Levene test. Categorical variables were compared using the Cochran-Armitage test for trends and the Pearson χ^2^ test or Fisher exact test for pairwise comparisons. To reduce the risk of a type I error in multiple comparisons, Bonferroni correction was applied where necessary.

Logistic regression was used to estimate the risk of severe complications with odds ratios and IQRs provided as risk estimates. Both univariate and multivariate logistic regression analyses were performed to identify the predictors of severe postoperative complications. Sensitivity analyses were conducted to assess the incidence of postoperative complications, excluding the learning curve effect (defined as a center experience of fewer than 40 cases) and separately evaluating outcomes for laparoscopic and robotic MIPD.^21^

Statistical significance was set at *P* <0.05, with a threshold of *P* <0.0167 for pairwise comparisons (after the Bonferroni correction). All analyses were performed using JMP Pro 17 and R Studio 2024.04.2, using the ggplot2 package for visualization.

## RESULTS

During the study period, 2257 minimally invasive pancreatic resections were reported in the IGOMIPS registry from 37 Italian centers. MIPD was planned in 816 patients (36.2%), and the procedure was completed in 770 patients (94.4%). An additional MIPD was performed in a patient who was initially scheduled for transduodenal ampullectomy. MIPD was performed at 25 IGOMIPS centers (67.5%), with a median number of reported MIPD per center of 14 (IQR: 5–37.5). Seven centers reported laparoscopic MIPD (28.0%), 5 reported both laparoscopic and robotic MIPD (20.0%), and 13 reported robotic MIPD (52.0%).

The MIPD cohort consisted of 546 robotic MIPD cases (70.8%) and 225 laparoscopic MIPD cases (19.2%). In the first year, 115 MIPD cases were enrolled in the registry, with a relatively balanced distribution between robotic (52 cases, 45.2%) and laparoscopic procedures (63 cases, 54.8%). In contrast, in the final year, 147 MIPD cases were recorded, of which 140 (95.2%) were robotic and 7 (4.8%) were laparoscopic. The shift toward robotic surgery became more pronounced over time, with a deflection point identified in case 357, after which there was a significantly lower probability of MIPD being performed laparoscopically (Area Under the Curve = 0.76146; *P* < 0.0001).

The reasons for not proceeding with the planned MIPD included the discovery of distant metastases (8 patients; 1.0%) and the decision to perform a different type of pancreatic resection in 38 patients (32/808; 3.9%), distal pancreatectomy in 2 patients (0.2%), total pancreatectomy in 28 patients (3.5%), and other pancreatic resections in 8 patients (1%). Ultimately, 771 MIPDs were performed, and owing to missing data, 426 of these procedures were analyzed in this study. The study flowchart is shown in Supplemental Figure 1 (Supplemental Digital Content Fig. 1, http://links.lww.com/SLA/F365). Preoperative characteristics and outcomes are presented in Table 1.

**Table 1:** Preoperative Characteristics and Outcomes of MIPD in the IGOMIPS Registry

	Results
Age (yr), median (IQR)	69 (61–75)
Sex (M), n (%)	236 (55.4)
BMI	
Median (IQR), kg/m^2^	24.5 (22.5–27)
≥30 kg/m^2^, n (%)	38 (8.9)
ASA score, n (%)	
I	22 (5.2)
II	209 (49.1)
III	189 (44.4)
IV	6 (1.4)
>II	195 (45.8)
Previous upper abdominal surgery, n (%)	156 (36.6)
Previous complex upper abdominal surgery, n (%)	1 (0.2)
Preoperative oncology treatments, n (%)	
Chemotherapy	22 (5.2)
Radiotherapy	3 (0.7)
Tumor vascular contact, n (%)	32 (7.5)
Main pancreatic duct diameter ≤3 mm, n (%)*	235 (55.4)
Common bile duct diameter ≤5 mm, n (%)	377 (88.4)
ISGPS complexity grading system, n (%)	
Grade A	22 (5.2)
Grade B	149 (35.0)
Grade C	255 (59.9)
ISGPS experience scoring system, n (%)	
Grade A	170 (39.9)
Grade B	161 (37.8)
Grade C	95 (22.3)
Approach, n (%)	
Laparoscopy	107 (25.1)
Hybrid laparoscopy	22 (5.2)
All laparoscopic approaches	129 (30.3)
Robotic	297 (69.7)
Operative time (min), mean ± SD	505.7 ± 109.8
Blood loss (mL), median (IQR)	200 (100–300)
Blood transfusion (mL), median (IQR)	0 (0-0)
Conversion, n (%)	
Due to intraoperative complications	7 (1.6)
All reasons	35 (8.4)
Firm pancreatic stump, n (%)	297 (72.1)
Pancreatic anastomosis, n (%)	
Pancreatojejunostomy	400 (94.5)
Pancreatogastrostomy	18 (4.3)
Duct occlusion	5 (1.2)
Length of hospital stay (d), median (IQR)	16 (10–27)
Postoperative complications, n (%)	
None	47 (11.0)
Grade 1	58 (13.6)
Grade 2	143 (33.6)
Grade 3a	82 (19.2)
Grade 3b	38 (8.9)
Grade 4a	21 (4.9)
Grade 4b	7 (1.6)
Grade 5	30 (7.0)
Grade >2	185 (43.4)
Failure-to-rescue, n (%)	30 (16.4)
POPF, n (%)	
Biochemical leak	51 (12.0)
Grade B/C	108 (25.4)
Grade C	23 (5.4)
Biliary leak, n (%)	40 (9.4)
Intestinal leak, n (%)	16 (3.8)
Chyle leak, n (%)	24 (5.6)
Delayed gastric emptying, n (%)	
Grade B/C	72 (16.9)
All grades	105 (24.6)
Post-pancreatectomy hemorrhage, n (%)	84 (19.7)
Drained abdominal fluid collections, n (%)	86 (20.2)
Reoperation, n (%)	65 (15.3)
Tumor type, n (%)	
Malignant tumor*	355 (88.8)
PDAC*	162 (40.5)
Lymph nodes, median (IQR)	
Examined*	24 (17–34)
Positive*	2 (1–5)

* Data missing in 2 cases for main pancreatic duct size, 26 cases for tumor type, 44 cases for examined lymph nodes, and 8 cases for positive nodes.

PDAC indicates pancreatic ductal adenocarcinoma.

Analysis of preoperative parameters identified a pancreatic duct diameter ≤3 mm as the only independent risk factor for developing severe postoperative complications [odds ratio = 2.24 (1.51–3.35), *P* < 0.0001].

### Validation of the International Study Group for Pancreatic Surgery Complexity Grading System

Table 2 presents the application of complexity scores to the IGOMIPS cohort. Table 3 provides additional baseline characteristics and relevant outcomes. Most MIPD in the IGOMIPS registry were classified as grade C (255/426; 59.9%), whereas a small minority were classified as A (22/426; 5.1%). Conversely, the majority of cases were managed by surgeon-center experience levels equivalent to ISGPS grades A (170/426; 39.9%) and B (161/426; 37.8%), with a small proportion of procedures performed by grade C surgeons at grade C centers (95/426; 22.3%). The distribution of surgical experience was relatively balanced across the population, with grade A at 144/426 (33.8%), grade B at 128/426 (30.0%), and grade C at 154/426 (36.2%). In contrast, there was a significant difference in the distribution of center experience levels: grade A centers accounted for 129/426 (30.3%), grade B for 202/426 (47.4%), and grade C for 95/426 (22.3%). Notably, grade C procedures were frequently performed by grade A centers (29.0%) or grade A surgeons (35.1%).

**Table 2:** ISGPS Complexity Scoring in the IGOMIPS Registry

	Complexity scoring			
	Grade A (n = 22)	Grade B (n = 149)	Grade C (n = 255)	*P*
ISGPS complexity parameters, n (%)				
Main pancreatic duct diameter ≤3 mm^*^	0	14 (9.4)	221 (87.4)	**<0.0001**
Common bile duct diameter ≤5 mm	0	135 (90.6)	242 (94.9)	**<0.0001**
Tumor vascular contact	0	0	32 (12.6)	**<0.0001**
BMI ≥30 kg/m^2^	0	0	38 (14.9)	**<0.0001**
Previous complex upper abdominal surgery	0	0	1 (0.4)	0.4440
ISGPS experience scoring system, n (%)				
Grade A	5 (22.7)	65 (43.6)	100 (39.2)	0.7360
Grade B	7 (31.8)	47 (31.5)	107 (42.0)	**0.0440**
Grade C	10 (45.5)	37 (24.8)	48 (18.8)	**0.0061**
ISGPS center classification				
Grade A	2 (9.1)	53 (35.6)	74 (29.0)	0.7972
Grade B	10 (45.5)	59 (39.6)	133 (52.2)	**0.0404**
Grade C	10 (45.5)	37 (24.8)	48 (18.8)	**0.0061**
ISGPS surgeon classification				
Grade A	4 (18.2)	50 (33.6)	90 (35.3)	0.2108
Grade B	6 (27.3)	42 (28.2)	80 (31.4)	0.4766
Grade C	12 (54.6)	57 (38.3)	85 (33.3)	0.0560
ISGPS center and surgeon classification, n (%)				
Center A	n = 2	n = 53	n = 74	
Surgeon A	1 (50.0)	38 (71.7)	64 (86.5)	**0.0217**
Surgeon B	1 (50.0)	14 (26.4)	8 (10.8)	**0.0108**
Surgeon C	0	1 (1.9)	2 (2.7)	0.7181
Center B	n = 10	n = 59	n = 26	
Surgeon A	3 (30.0)	12 (20.3)	26 (19.6)	0.5538
Surgeon B	5 (50.0)	28 (47.5)	72 (54.1)	0.4573
Surgeon C	2 (20.0)	19 (32.2)	35 (26.3)	0.7661
Center C	n = 10	n = 37	n = 48	
Surgeon A	0	0	0	NA
Surgeon B	0	0	0	NA
Surgeon C	10 (100.0)	37 (100.0)	48 (100.0)	NA

Bold values indicate significant *P.*

^*^ Data missing on 2 cases for main pancreatic duct size.

NA indicates not available.

**Table 3:** Additional Relevant Baseline Characteristics and Main Outcomes Based on ISGPS Complexity Scoring in the IGOMIPS Registry

	Complexity scoring			
	Grade A (n = 22)	Grade B (n = 149)	Grade C (n = 255)	*P*
Age (yr), median (IQR)	73.5 (67–79)	72 (64–77)	67 (57–74)	**<0.0001**
Sex (M), n (%)	13 (59.1)	85 (57.1)	138 (54.1)	0.5021
BMI (kg/m^2^), median (IQR)	25 (22.6–26)	24.2 (22.2–26.2)	24.6 (22.6–27.6)	0.0870
ASA score, n (%)				
I	0	6 (4.0)	16 (6.3)	0.1427
II	7 (31.8)	74 (49.7)	128 (50.2)	0.2740
III	14 (63.6)	65 (43.6)	110 (43.1)	0.2249
IV	1 (4.6)	4 (2.7)	1 (0.4)	**0.0228**
>II	15 (68.2)	69 (46.3)	111(43.5)	0.0803
Preoperative oncology treatments, n (%)				
Chemotherapy	0	7 (4.7)	15 (5.9)	0.2729
Radiotherapy	0	2 (1.3)	1 (0.4)	0.5309
Approach, n (%)				
Laparoscopy	3 (13.6)	36 (24.2)	68 (26.7)	0.2220
Hybrid laparoscopy	0	3 (2)	19 (7.5)	**0.0100**
All laparoscopic approaches	3 (13.6)	39 (26.2)	87 (34.1)	**0.0167**
Robotic	19 (86.4)	110 (73.8)	168 (65.9)	**0.0167**
Operative time, mean ± SD, min	482 ± 23.4	499.4 ± 9	511.4 ± 6.9	0.3337
Blood loss (mL), median (IQR)	200 (100–325)	177.5 (100–337.5)	200 (100–300)	0.8483
Blood transfusion (mL), median (IQR)	0 (0–0)	0 (0–0)	0 (0–0)	0.2575
Conversion, n (%)				
Due to intraoperative complications	0	2 (1.3)	5 (2)	0.4512
All reasons	0	13 (9.1)	22 (8.8)	0.4166
Firm pancreatic stump, n (%)	9 (40.9)	61 (43.3)	45 (18.1)	**<0.0001**
Pancreatic anastomosis, n (%)				
Pancreatojejunostomy	22 (100)	142 (96)	236 (93.3)	0.1085
Pancreatogastrostomy	0	4 (2.7)	14 (5.5)	0.0904
Duct occlusion	0	2 (1.4)	3 (1.2)	0.8381
Length of hospital stay (d), median (IQR)	15 (10–31.3)	14 (10–23)	17.5 (11–29)	0.1575
Postoperative complications, n (%)				
None	3 (13.6)	23 (15.4)	21 (8.2)	**0.0443**
Grade 1	2 (9.1)	24 (16.1)	32 (12.6)	0.6812
Grade 2	10 (45.5)	51 (34.2)	82 (32.2)	0.2820
Grade 3a	4 (18.2)	24 (16.1)	54 (21.2)	0.2854
Grade 3b	2 (9.1)	12 (8.1)	24 (9.4)	0.7272
Grade 4a	1 (4.6)	4 (2.7)	16 (6.3)	0.1844
Grade 4b	0	0	7 (2.8)	**0.0414**
Grade 5	0	11 (7.4)	19 (7.5)	0.4075
> Grade 2	7 (31.8)	54 (36.3)	124 (48.6)	**0.0091**
Failure-to-rescue, n (%)	0	11 (21.2)	19 (15.3)	0.9482
POPF, n (%)				
Biochemical leak	2 (9.1)	23 (15.4)	49 (19.2)	0.1590
Grade B/C	3 (13.6)	26 (17.5)	79 (31.0)	**0.0015**
Grade C	0	4 (2.7)	19 (7.5)	**0.0202**
Biliary leak, n (%)	0	15 (10.1)	25 (9.8)	0.3815
Intestinal leak, n (%)	1 (4.6)	5 (3.4)	10 (3.92)	0.9148
Chyle leak, n (%)	1 (4.6)	6 (4)	17 (6.7)	0.3082
Delayed gastric emptying, n (%)				
Grade B/C	5 (22.7)	19 (12.8)	48 (18.8)	0.4297
All grades	7 (31.8)	30 (20.1)	68 (26.7)	
Post-pancreatectomy hemorrhage, n (%)	4 (18.2)	26 (17.5)	54 (21.2)	0.4045
Abdominal fluid collections, n (%)				
Drained	3 (13.6)	21 (14.1)	62 (24.3)	**0.0148**
All	7 (31.8)	43 (28.9)	102 (40)	**0.0429**
Reoperation, n (%)	2 (9.1)	20 (13.4)	43 (16.9)	0.2154
Tumor type, n (%)				
Malignant tumor^*^	20 (100)	120 (87)	215 (88.8)	0.5865
PDAC^*^	11 (55)	67 (48.6)	84 (34.7)	**0.0035**
Lymph nodes, median (IQR)				
Examined^*^	(22.3–36.8)	23 (17–34)	23 (17–32)	0.0899
Positive^*^	6 (2–8)	2 (1–5)	2 (0–4)	**0.0303**

Bold values indicate significant *P*.

^*^ Data missing in 26 cases for tumor type, in 44 cases on a number of examined lymph nodes, and in 8 cases for positive.

PDAC indicates pancreatic ductal adenocarcinoma.

An increase in procedural complexity was significantly associated with a higher frequency of difficult factors, except for instances of previous complicated abdominal surgery. Interestingly, grade C complexity was correlated with a lower incidence of a hard pancreatic stump (18.1% vs 40.9% and 43.3% for grades A and B, respectively). The incidence of severe postoperative complications increased progressively from grade A to C, with rates of 31.8%, 36.3%, and 48.6%, respectively. Although postoperative mortality did not reach statistical significance, no deaths occurred in grade A MIPD, whereas mortality exceeded 7% in grades B and C.

Grade C procedures were associated with younger patients, fewer ASA IV cases, and greater reliance on laparoscopy over robotic approaches. Despite the higher complexity, ∼40% of grade C procedures are performed by grade A or grade A surgeons. This mismatch may contribute to the higher rates of severe postoperative complications, postoperative pancreatic fistula (POPF), grade C POPF, abdominal fluid collection, and the necessity for percutaneous drainage in grade C procedures.

Procedure difficulty was not associated with the length of hospital stay.

### Validation of the International Study Group for Pancreatic Surgery Surgeon and Center Experience Classification System

Table 4 summarizes the preoperative characteristics and outcomes based on surgeon and center experience. Table 5 provides additional baseline characteristics and relevant outcomes. MIPD in patients with vascular tumor contact was more frequent among those treated by grade C surgeons and centers. However, no significant differences were observed across experience levels regarding prior complicated abdominal surgery or an elevated BMI. Notably, the proportion of patients with a small pancreatic duct was lower at grade C experience levels, whereas the proportion of patients with small bile ducts remained consistent across the ISGPS experience levels.

**Table 4:** ISGPS Experience Scoring System in the IGOMIPS Registry

	Experience scoring						
	Grade A	Grade B	Grade C	*P*	pAB	pAC	pBC
ISGPS complexity parameters, n (%)							
Main pancreatic duct diameter ≤3 mm^*^	98 (57.7)	105 (65.2)	32 (34.4)	**0.0029**	0.1575	**0.0003**	**<0.0001**
Common bile duct diameter ≤5 mm	153 (90.0)	148 (91.9)	76 (80.0)	**0.0358**	0.5419	0.0227	**0.0053**
Tumor vascular contact	12 (7.1)	2 (1.2)	18 (19.0)	**0.0054**	**0.0116**	**0.0034**	**<0.0001**
BMI ≥30 kg/m^2^	11 (6.5)	23 (14.3)	4 (4.2)	0.9454	0.0192	0.5835	**0.0112**
Previous complex upper abdominal surgery	0	0	1 (1.1)	0.1256	NA	0.3585	0.3711
ISGPS center classification							
Grade A	129 (75.9)	0	0	**<0.0001**	**<0.0001**	**<0.0001**	NA
Grade B	41 (24.1)	161 (100.0)	0	0.4926	**<0.0001**	**<0.0001**	**<0.0001**
Grade C	0	0	95 (100.0)	**<0.0001**	NA	**<0.0001**	**<0.0001**
ISGPS surgeon classification							
Grade A	144 (84.7)	0	0	**<0.0001**	**<0.0001**	**<0.0001**	NA
Grade B	23 (13.5)	105 (65.2)	0	0.9491	**<0.0001**	**<0.0001**	**<0.0001**
Grade C	3 (1.8)	56 (34.8)	95 (100.0)	**<0.0001**	**<0.0001**	**<0.0001**	**<0.0001**
ISGPS center and surgeon classification, n (%)							
Center A	n = 129	n = 0	n = 0				
Surgeon A	103 (79.8)	0	0	NA	NA	NA	NA
Surgeon B	23 (17.8)	0	0	NA	NA	NA	NA
Surgeon C	3 (2.3)	0	0	NA	NA	NA	NA
Center B	n = 41	n = 161	n = 0				
Surgeon A	41 (100.0)	0	0	**<0.0001**	**<0.0001**	NA	NA
Surgeon B	0	105 (65.2)	0	**<0.0001**	**<0.0001**	NA	NA
Surgeon C	0	56 (34.8)	0	**<0.0001**	**<0.0001**	NA	NA
Center C	n = 0	n = 0	n = 95				
Surgeon A	0	0	0	NA	NA	NA	NA
Surgeon B	0	0	0	NA	NA	NA	NA
Surgeon C	0	0	95 (100.0)	NA	NA	NA	NA

Bold values indicate significant *P*.

^*^ Data missing on 2 cases for main pancreatic duct size.

NA indicates not available.

**Table 5:** Additional Relevant Baseline Characteristics and Main Outcomes Based on ISGPS Experience Scoring System in the IGOMIPS Registry

	Experience scoring						
	Grade A	Grade B	Grade C	*P*	pAB	pAC	pBC
Age (yr), median (IQR)	69 (60.8–75)	70 (61–76.5)	67 (61–74)	0.1710	0.4212	0.2411	0.0574
Sex (M), n (%)	92 (54.1)	90 (55.9)	54 (56.8)	0.6527	0.7445	0.6689	0.8834
BMI (kg/m^2^), median (IQR)	24.1 (22.2–26.3)	25.4 (23.2–27.8)	24.2 (22.3–26.6)	**0.0019**	**0.0008**	0.6084	**0.0146**
ASA score, n (%)							
I	9 (5.3)	12 (7.5)	1 (1.1)	0.2399	0.4205	0.1012	0.0351
II	95 (55.9)	90 (55.9)	24 (25.3)	**<0.0001**	0.9973	**<0.0001**	**<0.0001**
III	64 (37.7)	56 (34.8)	69 (72.6)	**<0.0001**	0.5879	**<0.0001**	**<0.0001**
IV	2 (1.2)	3 (1.9)	1 (1.1)	0.9760	0.6774	1.0000	1.0000
>II	66 (38.8)	59 (36.7)	70 (73.7)	**<0.0001**	0.6829	**<0.0001**	**<0.0001**
Preoperative oncology treatments, n (%)							
Chemotherapy	12 (7.1)	4 (2.5)	6 (6.3)	0.5448	0.0715	0.8177	0.1805
Radiotherapy	1 (0.6)	2 (1.2)	0	0.7222	0.6139	1.0000	0.5314
Approach, n (%)							
Laparoscopy	41 (24.1)	66 (41)	0	**0.0013**	**0.0010**	**<0.0001**	**<0.0001**
Hybrid laparoscopy	14 (8.2)	8 (5)	0	**0.0039**	0.2331	**0.0027**	0.0275
All laparoscopic approaches	55 (32.4)	74 (46)	0	**<0.0001**	**0.0112**	**<0.0001**	**<0.0001**
Robotic	115 (67.7)	87 (54)	95 (100)	**<0.0001**	**0.0112**	**<0.0001**	**<0.0001**
Operative time (min), mean±SD	513±8.2	474.4±8.4	546.1±11	**<0.0001**	**0.0021**	0.0199	**<0.0001**
Blood loss (mL), median (IQR)	200 (100–387.5)	200 (150–300)	150 (100–300)	**0.0033**	0.8501	**0.0025**	**0.0019**
Blood transfusion (mL), median (IQR)	0 (0–0)	0 (0–0)	0 (0–0)	**0.0030**	**0.0006**	0.3985	0.0232
Conversion, n (%)							
Due to intraoperative complications	4 (2.4)	3 (1.9)	0	0.1701	1.0000	0.3002	0.29718
All reasons	26(15.4)	8 (5)	1 (1.2)	**<0.0001**	**0.0019**	**0.0002**	0.167
Firm pancreatic stump, n (%)	41 (24.9)	112 (72.3)	31 (33.7)	0.1387	0.5566	0.1300	0.3234
Pancreatic anastomosis							
Pancreatico-jejunostomy	148 (87.6)	161 (100)	91 (97.9)	**<0.0001**	**<0.0001**	**0.0051**	0.1331
Pancreatico-gastrostomy	16 (9.5)	0	2 (2.2)	**0.0007**	**<0.0001**	0.0380	0.1331
Duct occlusion	5 (3)	0	0	**0.0160**	0.0610	0.1644	NA
Length of hospital stay (d), median (IQR)	15 (10–26)	17 (10–29)	18 (11–25.5)	0.3804	0.2813	0.2096	0.7943
Postoperative complications, n (%)							
None	0	0	47 (49.5)	**<0.0001**	NA	**<0.0001**	**<0.0001**
Grade 1	32 (18.8)	20 (12.4)	6 (6.3)	**0.0037**	0.1310	**0.0053**	0.1374
Grade 2	51 (30)	68 (42.2)	24 (25.3)	0.8077	0.0204	0.4117	**0.0063**
Grade 3a	38 (22.4)	34 (21.1)	10 (10.5)	**0.0302**	0.7855	**0.0165**	0.0300
Grade 3b	24 (14.1)	9 (5.6)	5 (5.3)	**0.0065**	**0.0096**	0.0384	1.0000
Grade 4a	8 (4.7)	12 (7.5)	1 (1.1)	0.3363	0.2944	0.1637	0.0351
Grade 4b	3 (1.8)	4 (2.5)	0	0.3809	0.7170	0.5550	0.3000
Grade 5	14 (8.2)	14 (8.7)	2 (2.1)	0.0980	0.8804	0.0582	0.0578
> Grade 2	87 (51.2)	73 (45.3)	25 (26.3)	**0.0002**	0.2883	**<0.0001**	**0.0025**
Failure-to-rescue, n (%)	14 (16.5)	14 (19.2)	2 (8)	0.5386	0.6568	0.5182	0.3458
POPF, n (%)							
Biochemical leak	29 (17.1)	41 (25.5)	4 (4.2)	**0.0464**	0.0612	**0.0018**	**<0.0001**
Grade B/C	51 (30)	42 (26.1)	15 (15.8)	**0.0139**	0.4286	**0.0103**	0.0557
Grade C	15 (8.8)	7 (4.3)	1 (1.1)	**0.0055**	0.1023	**0.0129**	0.2645
Biliary leak, n (%)	24 (14.1)	13 (8.1)	3 (3.2)	**0.0026**	0.0812	**0.0050**	0.1801
Intestinal leak, n (%)	4 (2.4)	11 (6.8)	1 (1.1)	0.9516	0.0643	0.6574	0.0355
Chyle leak, n (%)	12 (7.1)	9 (5.6)	3 (3.2)	0.1919	0.5838	0.2693	0.5435
Delayed gastric emptying, n (%)							
Grade B/C	25 (14.7)	34 (21.1)	13 (13.7)	0.9095	0.1276	0.8200	0.1378
All grades	36 (21.2)	53 (32.9)	16 (16.8)	0.8248	**0.0160**	0.3942	**0.0051**
Post-pancreatectomy hemorrhage, n (%)	41 (24.1)	37 (23)	6 (6.3)	**0.0014**	0.8076	**0.0002**	**0.0006**
Abdominal fluid collections, n (%)							
Drained abdominal fluid collection	43 (25.3)	27 (16.8)	16 (16.8)	0.0626	0.0577	0.1127	0.9881
All abdominal fluid collection	67 (39.4)	53 (32.9)	32 (33.7)	0.2784	0.2194	0.3553	0.9001
Reoperation, n (%)	36 (21.2)	22 (13.7)	7 (7.4)	**0.0021**	0.0724	**0.0035**	0.1247
Tumor type, n (%)							
Malignant tumor^*^	148 (87.1)	144 (90)	63 (90)	0.4183	0.4029	0.5252	1.0000
PDAC^*^	67 (39.4)	58 (36.3)	37 (52.9)	0.1446	0.5540	0.0561	0.0186
Lymph nodes, median (IQR)							
Examined^*^	23 (17–33)	20 (16–26)	38 (29–48.5)	**<0.0001**	**0.0031**	**<0.0001**	**<0.0001**
Positive^*^	1 (0–4)	1 (0–3)	3.5 (1–6)	**<0.0001**	0.5740	**<0.0001**	**<0.0001**

Bold values indicate significant *P*.

^*^ Data missing in 26 cases for tumor type, in 44 cases on a number of examined lymph nodes, and in 8 cases for positive.

NA indicates not available; PDAC, pancreatic ductal adenocarcinoma.

Patients treated by grade C surgeons demonstrated improved overall outcomes, including lower rates of severe postoperative complications, POPF, biliary leaks, postoperative hemorrhage (PPH), and reoperations. They also had a higher number of lymph nodes.

Surgeon and center experience were not associated with the length of hospital stay.

### Validation of the International Study Group for Pancreatic Surgery Patient Selection System

**Figure 1:**
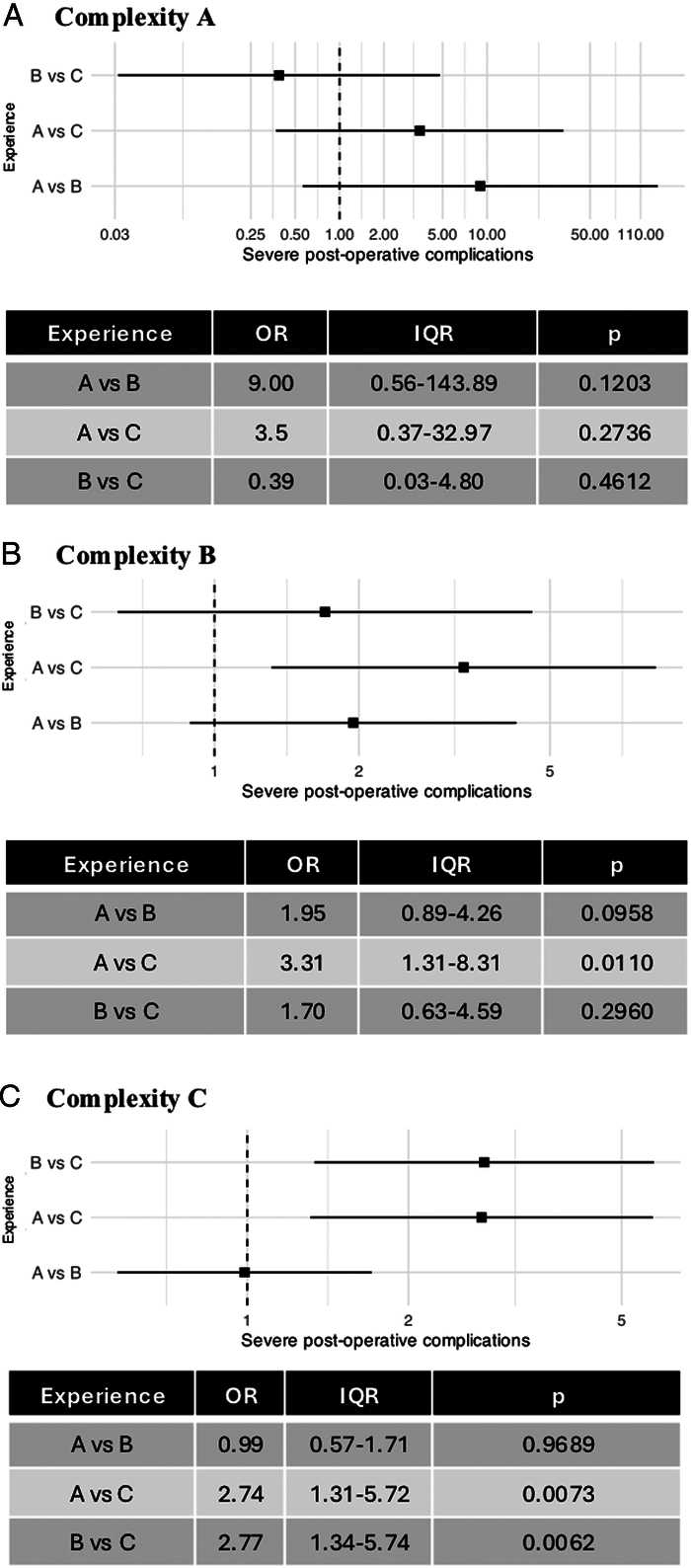
Risk of development of severe postoperative complication based on different grade of experience, stratified by procedure complexity.

Preoperative characteristics and outcomes, categorized by ISGPS experience level and stratified by procedural complexity, are presented in Supplemental Tables 1–3, (Supplemental Digital Content Tables 1–3, http://links.lww.com/SLA/F365). Figure 1 illustrates the risk of severe postoperative complications based on center experience and procedural complexity.

For grade A complexity, no significant differences in outcomes were observed across centers. Notably, there were no instances of 90-day mortality, grade C POPF, or biliary fistula.

For grade B complexity, the outcomes were comparable between grade B and C centers. However, procedures conducted at grade C centers resulted in fewer severe complications, lower 90-day mortality, reduced PPH rates, and fewer reoperations, along with improved POPF and conversion rates. Surgeries at grade B centers also demonstrated less blood loss and lower conversion rates than those at grade A centers.

For grade C complexity, the outcomes were similar between grade A and B centers. However, grade C centers exhibited fewer severe complications, lower rates of all grades of POPF, and decreased PPH. A higher proportion of patients experienced no postoperative complications in grade C centers.

### Impact of International Study Group for Pancreatic Surgery Experience Level on Mismatch Between Planned and Performed Pancreatic Resections

The ISGPS experience level was strongly correlated with the concordance between the planned and performed procedures. A different procedure than that declared the day before surgery was carried out for 30 out of 200 patients (15.0%) at the grade A ISGPS experience level, and for 5 out of 116 (3.0%) and 3 out of 98 (3.1%) patients with grade B and C ISGPS experience levels, respectively (*P* < 0.0001). The need to convert MIPD into total pancreatectomy was also associated with the ISGPS experience level, with grade A in 23 out of 200 (11.5%), grade B in 2 out of 166 (1.2%), and grade C in 3 out of 98 (3.1%) patients (*P* = 0.0005). It is noteworthy that differences in the need for either a procedure other than MIPD or specifically a total pancreatectomy were significant between grade A ISGPS experience level and grade B/C, but not between grade B and C experience levels.

### Sensitivity Analyses

To exclude the impact of the feasibility learning curve on the incidence of severe postoperative complications, the first 40 MIPD cases per center were excluded from the analysis. Based on this criterion, 17 of 25 centers (68.0%) did not complete the feasibility learning curve when they enrolled their first case in the IGOMIPS registry. Four of these centers (23.6%) completed the learning curve during their participation in the registry. After excluding cases performed before reaching this time point, the incidence of severe postoperative complications increased with procedural complexity (grade A 27.8%; grade B, 30.1%; and grade C, 45.5%; *P* = 0.0138).

To assess the impact of surgical technique on the incidence of severe postoperative complications, the outcomes of laparoscopic and robotic MIPD were analyzed separately. An improvement in the rate of severe postoperative complications was observed for robotic MIPD (grade A 26.3%; grade B, 31.8%; grade C, 42.3%; *P* = 0.0138), but not for laparoscopic MIPD (grade A 66.7%; grade B, 48.7%; grade C, 60.9%; *P* = 0.3383). This discrepancy may explain the shift from laparoscopic to robotic MIPD observed in our registry.

## DISCUSSION

ISGPS recently introduced a complexity grading system for MIPD to standardize and promote the safe adoption of this approach. This system is based on the well-established literature and incorporates factors strongly associated with procedural difficulty.^11^ Although prospective studies are ideal for validation, data from the prospective IGOMIPS registry (2019–2023) enable a timely, albeit retrospective, assessment of its clinical utility. This analysis also provides insights into potential refinements of the scoring system based on real-world outcomes.

This study validated the ability of the ISGPS score to predict MIPD outcomes based on procedural complexity. This highlights the importance of aligning surgeons' and centers’ experience with procedural complexity to minimize complications and improve outcomes. Although high-experience centers (grade C) performed better overall, the higher rates of severe complications in grade C procedures performed at grade A centers suggest that training and procedure selection should be optimized for more complex cases.

Notably, 4% of patients scheduled for MIPD underwent a different procedure, often a total pancreatectomy. While total pancreatectomy eliminates the risks associated with POPF, it presents unique challenges in a minimally invasive setting.^22–24^ The need for total pancreatectomy may reflect an unfavorable tumor location or more advanced disease, requiring technical modifications. Centers pursuing MIPD should be prepared for alternative procedures and have the flexibility to adjust them intraoperatively.

The ISGPS is not the only grading system for MIPD complexity. The PD-ROBOSCORE, developed specifically for robotic PD, focuses on procedural difficulty without accounting for surgeon or center experience.^10^ PD-ROBOSCORE includes 6 parameters, 3 of which overlap with the ISGPS: high BMI, small pancreatic duct, and vascular tumor contact. However, PD-ROBOSCORE emphasizes anatomic challenges, such as uncinate process tumors and variant hepatic arteries, while the ISGPS score includes previous complicated abdominal surgery and small bile ducts. Despite these differences, both systems underscore the complexity of MIPD.^10,11^

Among all ISGPS parameters, a small pancreatic duct size was a predictor of severe postoperative complications in the IGOMIPS registry. Remarkably, despite the fact that 40% of grade C procedures were performed by grade A or grade A centers, grade C MIPD at grade C centers had lower rates of severe complications, emphasizing the importance of both individual and institutional experience in complex cases.

Severe postoperative complications are pivotal outcomes in PD, as they prolong hospital stays,^25^ increase costs and mortality rates,^25,26^ delay or prevent adjuvant oncologic treatments,^27^ and adversely affect overall and disease-free survival.^28^ Although the search for an ideal outcome metric in PD continues and holistic scoring systems are being proposed,^29^ the reduction of postoperative complications remains the cornerstone of successful pancreatic surgery.

In the IGOMIPS registry, MIPD did not significantly reduce the incidence of severe postoperative complications compared with the rates typically seen with open PD. These findings align with those of 2 recent randomized controlled trials that compared MIPD with open PD.^30,31^ However, outcomes markedly improved when MIPD was performed by expert surgeons at high-volume centers, underscoring the importance of both surgeon and center volumes.

Interestingly, the relevance of previous complicated upper abdominal surgery as a difficulty parameter may be overstated, as 0.2% of the patients met this criterion. Surgeons may view this condition as a contraindication rather than a challenge for MIPD, reflecting improved patient selection.

Patient selection remains critical, particularly with regard to duct size. A high proportion of MIPDs were performed on patients with small pancreatic and bile ducts, which poses significant technical challenges. In a minimally invasive setting, these challenges are amplified, with some cases appearing straightforward during resection but proving difficult during reconstruction.^32^

Vein resection and reconstruction increase the risk of severe complications in open PD;^33^ data on vascular resections in MIPD are limited. Although many centers consider this a contraindication for MIPD, recent studies have shown its feasibility at high-volume centers in select patients.^34,35^ The new REDISCOVER guidelines and 2023 international consensus guidelines on robotic pancreatic surgery support minimally invasive approaches for borderline resectable cases, emphasizing patient selection and surgeon experience.^36–38^ The high conversion rates, particularly for cases involving vein resection, highlight the increased complexity of MIPD.^30^

This study found that 5% of MIPD cases were classified as low complexity. While this highlights the critical role of patient selection, it also raises the question of how many “easy” cases can truly be identified in PD, a procedure inherently complex by nature. This consideration likely extends to open PD as well, where poor outcomes have consistently been linked to low-volume centers, often regarded as a comprehensive quality metric.^39^ Thus, the debate on MIPD complexity may also apply to open PD, potentially revealing outcome-related factors beyond the volume.

Laparoscopy is more frequently used in grade C procedures. This may be due to the early adoption of laparoscopic MIPD, resulting in some surgeons being more comfortable with laparoscopy than with robotics in complex cases, or due to limited access to robotic systems. In addition, the lack of specialized robotic tools for efficient dissection may have contributed to this, as such tools are more readily available for laparoscopy. It is also possible that some surgeons were unaware of the complexity of MIPD because of the absence of a complexity score. This aligns with the finding that less experienced centers often handle a higher proportion of complex cases, while more experienced centers tend to select easier cases, as seen in the validation of the PD-ROBOSCORE.^10^ Ultimately, as surgeons complete their learning curve, the proportion of difficult cases decreases across all centers, indicating that mastering MIPD involves not only technical skills but also refining patient selection. This reflects the principle that the clinical benefits of minimally invasive surgery decrease with the complexity of the procedures.

Over time, the use of laparoscopy has gradually decreased in the IGOMIPS registry, as already shown in the E-MIPS registry.^40^ While there is no definitive evidence that robotic assistance improves MIPD outcomes and concerns persist regarding costs and accessibility, the clinical implementation trends show that robotic assistance was preferred by Italian surgeons.^40,41^

This study did not demonstrate a reduction in hospital stay length based on procedural difficulty or surgeon/center experience, despite the higher rates of severe postoperative complications in grade C procedures and fewer complications in grade C centers. Several factors may explain the contradictory findings. First, while enhanced recovery after surgery protocols can reduce hospital stays, their use in the IGOMIPS registry is not mandatory.^42^ Second, hospital stay lengths can vary geographically. For instance, a study found a 4-fold difference in stay length after pancreatectomy between the USA and Japan, with Italian hospitals—mostly public—reporting lengths similar to Japan.^43,44^ Third, most pancreatic resections in Italy are centralized in select centers and require long-distance travel for patients upon discharge. Finally, a mismatch between procedure difficulty and surgeon/center expertise may have obscured the impact of the ISGPS score on hospital stay duration.

This study had several limitations. The bile duct size and vascular tumor contact were not prospectively recorded in the IGOMIPS registry. Although small bile ducts are linked to difficult anastomosis and vascular tumor contact with vascular resections, surrogate markers were used to estimate these factors. In addition, being part of a registry may have led some centers to broaden MIPD indications to increase patient enrollment, contributing to the observed high-complexity cases. As a result, the final validation of the ISGPS complexity grading system will require prospective studies explicitly designed for this purpose. While such studies might yield more selective findings compared with the broader scope of registry-based analyses, they will provide critical insights into the true clinical utility of MIPD. These insights will be pivotal in ensuring the continued safe and effective global implementation of MIPD. In fact, given the growing demand for minimally invasive pancreatic surgery, the inherent complexity of MIPD necessitates a cautious and evidence-based approach. In this context, the progressive adoption of increasingly complex cases should be guided by the acquisition of both practical skills and cognitive knowledge to ensure optimal patient outcomes.

## CONCLUSIONS

This study provides the first clinical validation of the ISGPS complexity score for MIPD. Although further refinements are likely to be experienced as the system grows, the ISGPS score represents a vital tool for promoting safe MIPD adoption and enabling meaningful comparisons between centers and techniques, including open PD.

## Supplementary Material

## References

[1] Sánchez-Velázquez P Muller X Malleo G. Benchmarks in pancreatic surgery: a novel tool for unbiased outcome comparisons. Ann Surg. 2019;270:211–218. 30829701 10.1097/SLA.0000000000003223

[2] Sabogal JC Conde Monroy D Rey Chaves CE. Delayed gastric emptying after pancreatoduodenectomy: an analysis of risk factors. Updates Surg. 2024;76:1247–1255. 38598061 10.1007/s13304-024-01795-6 PMC11341576

[3] Hobeika C Pfister M Geller D. Recommendations on robotic hepato-pancreato-biliary surgery. the Paris jury-based consensus conference. Ann Surg. 2025;281:136–153. 38787528 10.1097/SLA.0000000000006365

[4] Asbun HJ Moekotte AL Vissers FL. The Miami international evidence-based guidelines on minimally invasive pancreas resection. Ann Surg. 2020;271:1–14. 31567509 10.1097/SLA.0000000000003590

[5] Abu Hilal M van Ramshorst TME Boggi U. The Brescia internationally validated European guidelines on minimally invasive pancreatic surgery (EGUMIPS). Ann Surg. 2024;279:45–57. 37450702 10.1097/SLA.0000000000006006 PMC10727198

[6] van Hilst J de Rooij T Bosscha K. Laparoscopic versus open pancreatoduodenectomy for pancreatic or periampullary tumours (LEOPARD-2): a multicentre, patient-blinded, randomised controlled phase 2/3 trial. Lancet Gastroenterol Hepatol. 2019;4:199–207. 30685489 10.1016/S2468-1253(19)30004-4

[7] Bhandare MS Parray A Chaudhari VA. Minimally invasive surgery for pancreatic cancer-are we there yet? A narrative review. Chin Clin Oncol. 2022;11:3. 35255692 10.21037/cco-21-131

[8] Napoli N Kauffmann EF Menonna F. Indications, technique, and results of robotic pancreatoduodenectomy. Updates Surg. 2016;68:295–305. 27614901 10.1007/s13304-016-0387-7

[9] Zwart MJW van den Broek B de Graaf N. The feasibility, proficiency, and mastery learning curves in 635 robotic pancreatoduodenectomies following a multicenter training program: “standing on the shoulders of giants”. Ann Surg. 2023;278:e1232–e1241. 37288547 10.1097/SLA.0000000000005928 PMC10631507

[10] Napoli N Cacace C Kauffmann EF. The PD-ROBOSCORE: a difficulty score for robotic pancreatoduodenectomy. Surgery. 2023;173:1438–1446. 36973127 10.1016/j.surg.2023.02.020

[11] Barreto SG Strobel O Salvia R. Complexity and experience grading to guide patient selection for minimally-invasive pancreatoduodenectomy: An ISGPS consensus. Ann Surg. 2024;Published online July 22, 2024. 10.1097/SLA.0000000000006454 39034920

[12] Zerbi A Capretti G Napoli N. The Italian National Registry for Minimally Invasive Pancreatic Surgery: an initiative of the Italian group of minimally invasive pancreas surgery (IGoMIPS). Updates Surg. 2020;72:379–385. 32468424 10.1007/s13304-020-00808-4

[13] Boggi U Donisi G Napoli N. Prospective minimally invasive pancreatic resections from the IGOMIPS registry: a snapshot of daily practice in Italy on 1191 between 2019 and 2022. *Updates Surg.* 2024;76:327-328. 10.1007/s13304-023-01709-y PMC10805981 38041779

[14] World Medical Association. World Medical Association Declaration of Helsinki: ethical principles for medical research involving human subjects. JAMA. 2013;310:2191–2194. 24141714 10.1001/jama.2013.281053

[15] von Elm E Altman DG Egger M. Strengthening the Reporting of Observational studies in Epidemiology Initiative. The Strengthening the reporting of observational studies in epidemiology statement: guidelines for reporting observational studies. Int J Surg. 2014;12:1495–1499. 25046131 10.1016/j.ijsu.2014.07.013

[16] Dindo D Demartines N Clavien PA. Classification of surgical complications: a new proposal with evaluation in a cohort of 6336 patients and results of a survey. Ann Surg. 2004;240:205–213. 15273542 10.1097/01.sla.0000133083.54934.ae PMC1360123

[17] Bassi C Marchegiani G Dervenis C. The 2016 update of the International Study Group (ISGPS) definition and grading of postoperative pancreatic fistula: 11 tears after. Surgery. 2017;161:584–591. 28040257 10.1016/j.surg.2016.11.014

[18] Wente MN Veit JA Bassi C. Postpancreatectomy hemorrhage (PPH): an International Study Group of Pancreatic Surgery (ISGPS) definition. Surgery. 2007;142:20–25. 17629996 10.1016/j.surg.2007.02.001

[19] Wente MN Bassi C Dervenis C. Delayed gastric emptying (DGE) after pancreatic surgery: a suggested definition by the International Study Group of Pancreatic Surgery (ISGPS). Surgery. 2007;142:761–768. 17981197 10.1016/j.surg.2007.05.005

[20] Besselink MG van Rijssen LB Bassi C. Definition and classification of chyle leak after pancreatic operation: a consensus statement by the International Study Group on Pancreatic Surgery. Surgery. 2017;161:365–372. 27692778 10.1016/j.surg.2016.06.058

[21] Jones LR Zwart MJW de Graaf N. Learning curve stratified outcomes after robotic pancreatoduodenectomy: International multicenter experience. Surgery. 2024;176:1721–1729. 39164152 10.1016/j.surg.2024.05.044

[22] Boggi U Palladino S Massimetti G. Laparoscopic robot-assisted versus open total pancreatectomy: a case-matched study. Surg Endosc. 2015;29:1425–1432. 25159652 10.1007/s00464-014-3819-9

[23] Kauffmann EF Napoli N Genovese V. Feasibility and safety of robotic-assisted total pancreatectomy: a pilot western series. Updates Surg. 2021;73:955–966. 34009627 10.1007/s13304-021-01079-3 PMC8184722

[24] Scholten L Klompmaker S Van Hilst J. Outcomes after minimally invasive versus open total pancreatectomy: a pan-European propensity score matched study. Ann Surg. 2023;277:313–320. 34261885 10.1097/SLA.0000000000005075

[25] Wang J Ma R Eleftheriou P. Health economic implications of complications associated with pancreaticoduodenectomy at a University Hospital: a retrospective cohort cost study. HPB (Oxford). 2018;20:423–431. 29248401 10.1016/j.hpb.2017.11.001

[26] Tamirisa NP Parmar AD Vargas GM. Relative contributions of complications and failure to rescue on mortality in older patients undergoing pancreatectomy. Ann Surg. 2016;263:385–391. 25563871 10.1097/SLA.0000000000001093 PMC5404345

[27] Wu W He J Cameron JL. The impact of postoperative complications on the administration of adjuvant therapy following pancreaticoduodenectomy for adenocarcinoma. Ann Surg Oncol. 2014;21:2873–2881. 24770680 10.1245/s10434-014-3722-6 PMC4454347

[28] Lubrano J Bachelier P Paye F. Severe postoperative complications decrease overall and disease free survival in pancreatic ductal adenocarcinoma after pancreaticoduodenectomy. Eur J Surg Oncol. 2018;44:1078–1082. 29685757 10.1016/j.ejso.2018.03.024

[29] Partelli S Fermi F Fusai GK. The value of textbook outcome in benchmarking pancreatoduodenectomy for nonfunctioning pancreatic neuroendocrine tumors. Ann Surg Oncol. 2024;31:4096–4104. 38461463 10.1245/s10434-024-15114-1

[30] Klotz R Mihaljevic AL Kulu Y. Robotic versus open partial pancreatoduodenectomy (EUROPA): a randomised controlled stage 2b trial. Lancet Reg Health Eur. 2024;39:100864. 38420108 10.1016/j.lanepe.2024.100864 PMC10899052

[31] Liu Q Li M Gao Y. Effect of robotic versus open pancreaticoduodenectomy on postoperative length of hospital stay and complications for pancreatic head or periampullary tumours: a multicentre, open-label randomised controlled. Lancet Gastroenterol Hepatol. 2024;9:428–437. 38428441 10.1016/S2468-1253(24)00005-0

[32] Schuh F Mihaljevic AL Probst P. A simple classification of pancreatic duct size and texture predicts postoperative pancreatic fistula: a classification of the International Study Group of Pancreatic Surgery. Ann Surg. 2023;277:e597–e608. 33914473 10.1097/SLA.0000000000004855 PMC9891297

[33] Kleive D Sahakyan MA Berstad AE. Trends in indications, complications and outcomes for venous resection during pancreatoduodenectomy. Br J Surg. 2017;104:1558–1567. 28815556 10.1002/bjs.10603

[34] Kauffmann EF Napoli N Ginesini M. Tips and tricks for robotic pancreatoduodenectomy with superior mesenteric/portal vein resection and reconstruction. Surg Endosc. 2023;37:3233–3245. 36624216 10.1007/s00464-022-09860-0 PMC10082118

[35] Napoli N Kauffmann EF Ginesini M. Robotic versus open pancreatoduodenectomy with vein resection and reconstruction: a propensity score-matched analysis. Ann Surg Open. 2024;5:e409. 38911629 10.1097/AS9.0000000000000409 PMC11191888

[36] Boggi U Kauffmann E Napoli N. REDISCOVER international guidelines on the perioperative care of surgical patients with borderline-resectable and locally advanced pancreatic cancer. Ann Surg. 2024;280:56–65. 38407228 10.1097/SLA.0000000000006248 PMC11161250

[37] Boggi U Kauffmann EF Napoli N. REDISCOVER guidelines for borderline-resectable and locally advanced pancreatic cancer: management algorithm, unanswered questions, and future perspectives. Updates Surg. 2024;76:1573–1591. 38684573 10.1007/s13304-024-01860-0 PMC11455680

[38] Liu R Abu Hilal M Besselink MG. International consensus guidelines on robotic pancreatic surgery in 2023. Hepatobiliary Surg Nutr. 2024;13:89–104. 38322212 10.21037/hbsn-23-132 PMC10839730

[39] Ratnayake B Pendharkar SA Connor S. Patient volume and clinical outcome after pancreatic cancer resection: A contemporary systematic review and meta-analysis. Surgery. 2022;172:273–283. 35034796 10.1016/j.surg.2021.11.029

[40] Emmen AMLH de Graaf N Khatkov IE. Implementation and outcome of minimally invasive pancreatoduodenectomy in Europe: a registry-based retrospective study - a critical appraisal of the first 3 years of the E-MIPS registry. Int J Surg. 2024;110:2226–2233. 38265434 10.1097/JS9.0000000000001121 PMC11019999

[41] Emmen AMLH Zwart MJW Khatkov IE. Robot-assisted versus laparoscopic pancreatoduodenectomy: a pan-European multicenter propensity-matched study. Surgery. 2024;175:1587–1594. 38570225 10.1016/j.surg.2024.02.015

[42] Hurley MP Schoemaker L Morton JM. Geographic variation in surgical outcomes and cost between the United States and Japan. Am J Manag Care. 2016;22:600–607. 27662222

[43] Kuemmerli C Tschuor C Kasai M. Impact of enhanced recovery protocols after pancreatoduodenectomy: meta-analysis. Br J Surg. 2022;109:256–266. 35037019 10.1093/bjs/znab436

[44] Boggi U Signori S De Lio N. F. Feasibility of robotic pancreaticoduodenectomy. Br J Surg. 2013;100:917–925. 23640668 10.1002/bjs.9135

