# Supplementary material for: External Validation of the International Study Group for Pancreatic Surgery Complexity Grading System for Minimally Invasive Pancreatoduodenectomy: Insights From the IGOMIPS Registry
Source: Ann Surg. 2024 Dec 18;284(1):108–18. doi: 10.1097/SLA.0000000000006612 (PMC13258101; doi:10.1097/SLA.0000000000006612)
Supplement: Supplementary file 1 [file sla-284-108-s001.docx]

**Supplementary table 1 -** Preoperative and postoperative results classified for the different ISGPS experience groups considering only ISGPS complexity grade A procedures

|  | **Experience Scoring** | | |  |  |  |  |
| --- | --- | --- | --- | --- | --- | --- | --- |
|  | **Grade A** | **Grade B** | **Grade C** | **p** | **pAB** | **pAC** | **pBC** |
| **ISGPS complexity parameters**, n (%) |  |  |  |  |  |  |  |
| Main pancreatic duct diameter ≤3 mm | 0 | 0 | 0 | NA | NA | NA | NA |
| Common bile duct diameter ≤5 mm | 0 | 0 | 0 | NA | NA | NA | NA |
| Tumor vascular contact | 0 | 0 | 0 | NA | NA | NA | NA |
| BMI ≥30 kg/m^2^ | 0 | 0 | 0 | NA | NA | NA | NA |
| Previous complex upper abdominal surgery | 0 | 0 | 0 | NA | NA | NA | NA |
|  |  |  |  |  |  |  |  |
| **ISGPS center classification**, n (%) |  |  |  |  |  |  |  |
| Grade A | 2 (40.0%) | 0 | 0 | **0.0218** | 0.1515 | 0.0952 | NA |
| Grade B | 3 (60.0%) | 7 (100.0%) | 0 | **0.0045** | 0.1515 | **0.0220** | **<0.0001** |
| Grade C | 0 | 0 | 10 (100.0%) | **<0.0001** | NA | **0.0003** | **<0.0001** |
|  |  |  |  |  |  |  |  |
| **ISGPS surgeon classification**, n (%) |  |  |  |  |  |  |  |
| Grade A | 4 (80.0%) | 0 | 0 | **0.0006** | **0.0101** | **0.0037** | NA |
| Grade B | 1 (20.0%) | 5 (71.4%) | 0 | **0.1540** | 0.2424 | 0.3333 | **0.0034** |
| Grade C | 0 | 2 (28.6%) | 10 (100.0%) | **<0.0001** | 0.4697 | **0.0003** | **0.0034** |
|  |  |  |  |  |  |  |  |
| **ISGPS center and surgeon classification,** n (%) |  |  |  |  |  |  |  |
| Center A |  |  |  |  |  |  |  |
| Surgeon A | 1 (50.0%) | 0 | 0 | NA | NA | NA | NA |
| Surgeon B | 1 (50.0%) | 0 | 0 | NA | NA | NA | NA |
| Surgeon C | 0 | 0 | 0 | NA | NA | NA | NA |
|  |  |  |  |  |  |  |  |
| Center B |  |  |  |  |  |  |  |
| Surgeon A | 3 (100.0%) | 0 | 0 | **0.0016** | **0.0083** | NA | NA |
| Surgeon B | 0 | 5 (71.4%) | 0 | **0.0384** | 0.1667 | NA | NA |
| Surgeon C | 0 | 2 (28.6%) | 0 | 0.3006 | 1.0000 | NA | NA |
|  |  |  |  |  |  |  |  |
| Center C |  |  |  |  |  |  |  |
| Surgeon A | 0 | 0 | 0 | NA | NA | NA | NA |
| Surgeon B | 0 | 0 | 0 | NA | NA | NA | NA |
| Surgeon C | 0 | 0 | 10 (100.0%) | NA | NA | NA | NA |
|  |  |  |  |  |  |  |  |
| Age, median (IQR), years | 77 (63.5-82.5) | 75 (72-79) | 67.5 (58.3-76.3) | 0.1511 | 1.000 | 0.2966 | 0.0563 |
| Male gender, n (%) | 4 (80.0%) | 3 (42.9%) | 6 (60.0%) | 0.6021 | 0.2929 | 0.6004 | 0.6372 |
| BMI, median (IQR), kg/m^2^ | 25.8 (22.5-26.4) | 25.7 (22.5-26.6) | 24.5 (22-26.1) | 0.7295 | 0.9447 | 0.6668 | 0.4634 |
| ASA score, n (%)  I  II  III  IV  >II | 0  3 (60.0%)  2 (40.0%)  0  2 (40.0%) | 0  2 (28.6%)  4 (57.1%)  1 (14.3%)  5 (71.4%) | 0  2 (20.0%)  8 (80.0%)  0  8 (80.0%) | NA  0.1352  0.1156  0.7695  0.1352 | NA  0.5581  1.0000  1.0000  0.5581 | NA  0.2507  0.2507  NA  0.2507 | NA  1.0000  0.5928  0.4118  1.0000 |
| Pre-operative oncology treatments, n (%)  Chemotherapy  Radiotherapy | 0  0 | 0  0 | 0  0 | NA  NA | NA  NA | NA  NA | NA  NA |
| Approach, n (%)  Laparoscopy  Hybrid laparoscopy  All laparoscopic approaches  Robotic | 2 (40.0%)  0  2 (40.0%)  3 (60.0%) | 1 (14.3%)  0  1 (14.3%)  6 (85.7%) | 0  0  0  10 (100.0%) | **0.0358**  NA  **0.0358**  **0.0358** | 0.5227  NA  0.5227  0.5227 | 0.0952  NA  0.0952  0.0952 | 0.4118  NA  0.4118  0.4118 |
| Operative time, mean$\pm$SD, min | 477$\pm$38.5 | 409.9$\pm$32.5 | 535$\pm$27.2 | **0.0276** | 0.2904 | 0.2295 | **0.0041** |
| Blood loss, median (IQR), mL | 400 (225-550) | 150 (100-200) | 180 (50-325) | 0.0765 | 0.0335 | 0.0839 | 0.6923 |
| Blood transfusion, median (IQR), mL | 0 (0-0) | 0 (0-0) | 0 (0-0) | 0.7174 | 0.4990 | 0.5716 | 0.9306 |
| Conversion, n (%)  Due to intraoperative complications  All reasons | 0  0 | 0  0 | 0  0 | NA  NA | NA  NA | NA  NA | NA  NA |
| Firm pancreatic stump, n (%) | 2 (40.0%) | 4 (57.1%) | 3 (30.0%) | 0.5679 | 1.0000 | 1.0000 | 0.3500 |
| Pancreatic anastomosis, n (%)  Pancreatico-jejunostomy  Pancreatico-gastrostomy  Duct occlusion | 5 (100.0%)  0  0 | 7 (100.0%)  0  0 | 10 (100.0%)  0  0 | NA  NA  NA | NA  NA  NA | NA  NA  NA | NA  NA  NA |
| Length of hospital stay, median (IQR), days | 18 (9.5-29.5) | 14 (9-43) | 20 (12.3-31.3) | 0.7962 | 0.8703 | 0.6601 | 0.6015 |
| Postoperative complications, n (%)  None  Grade 1  Grade 2  Grade 3a  Grade 3b  Grade 4a  Grade 4b  Grade 5  > Grade 2 | 0  1 (20.0%)  1 (20.0%)  3 (60.0%)  0  0  0  0  3 (60.0%) | 0  0  6 (85.7%)  0  0  1 (14.3%)  0  0  1 (14.3%) | 3 (30.0%)  1 (10.0%)  3 (30.0%)  1 (10.0%)  2 (20.0%)  0  0  0  3 (30.0%) | 0.0696  0.6711  0.8831  **0.0428**  0.1488  0.7695  NA  NA  0.3590 | NA  0.4167  0.0720  0.0455  NA  1.0000  NA  NA  0.2222 | 0.5055  1.0000  1.0000  0.0769  0.5238  NA  NA  NA  0.3287 | 0.2279  1.0000  0.0498  1.0000  0.4853  0.4118  NA  NA  0.6029 |
| Failure-to-rescue, n (%) | 0 | 0 | 0 | NA | NA | NA | NA |
| Post-operative pancreatic fistula, n (%)  Biochemical leak  Grade B/C  Grade C | 1 (20.0%)  0  0 | 1 (14.3%)  1 (14.3%)  0 | 0  2 (20.0%)  0 | 0.1742  0.3022  NA | 1.0000  1.0000  NA | 0.3333  0.5238  NA | 0.4118  1.0000  NA |
| Biliary leak, n (%) | 0 | 0 | 0 | NA | NA | NA | NA |
| Intestinal leak, n (%) | 0 | 0 | 1 (10.0%) | 0.3191 | NA | 1.0000 | 1.0000 |
| Chyle leak, n (%) | 0 | 1 (14.3%) | 0 | 0.7695 | 1.0000 | NA | 0.4118 |
| Delayed gastric emptying, n (%)  Grade B/C  All grades | 1 (20.0%)  1 (20.0%) | 2 (28.6%)  4 (57.1%) | 2 (20.0%)  2 (20.0%) | 0.9304  0.7333 | 1.0000  0.2929 | 1.0000  1.0000 | 1.0000  0.1618 |
| Post-pancreatectomy hemorrhage, n (%) | 1 (20.0%) | 1 (14.3%) | 2 (20.0%) | 0.9495 | 1.0000 | 1.0000 | 1.0000 |
| Abdominal fluid collections, n (%)  Drained abdominal fluid collection  All abdominal fluid collection | 0  0 | 1 (14.3%)  2 (28.6%) | 2 (20.0%)  5 (50.0%) | 0.3022  **0.0493** | 1.0000  0.4697 | 0.5238  0.1009 | 1.0000  0.6221 |
| Reoperation, n (%) | 0 | 0 | 2 (20.0%) | 0.1488 | NA | 0.5238 | 0.4853 |
| Tumor type, n (%)  Malignant tumor*  PDAC* | 5 (100.0%)  1 (20.0%) | 7 (100.0%)  5 (71.4%) | 8 (100.0%)  5 (62.5%) | NA  0.1824 | NA  0.2424 | NA  0.2657 | NA  1.0000 |
| Lymph nodes, median***** (IQR) Examined  Positive* | 28 (16.5-34.5)  0 (0-4) | 23 (22-34)  6 (1-8) | 36 (27.5-40.5)  2.5 (0.3-7.5) | 0.1912  0.2397 | 0.9351  0.1150 | 0.1643  0.2553 | 0.1318  0.6395 |

***** Data missing on 2 cases for tumor type, on 17 cases on number of examined lymph nodes, and on 8 cases for positive; PDAC: Pancreatic Ductal Adenocarcinoma; **Bold= significative p**

**Supplementary table 2** – Preoperative and postoperative results classified for the different ISGPS experience groups considering only ISGPS complexity grade B procedures

|  | **Experience Scoring** | | |  |  |  |  |
| --- | --- | --- | --- | --- | --- | --- | --- |
|  | **Grade A** | **Grade B** | **Grade C** | **p** | **pAB** | **pAC** | **pBC** |
| **ISGPS complexity parameters**, n (%) |  |  |  |  |  |  |  |
| Main pancreatic duct diameter ≤3 mm***** | 9 (13.9%) | 4 (8.5%) | 1 (2.7%) | 0.0614 | 0.5521 | 0.0892 | 0.3781 |
| Common bile duct diameter ≤5 mm | 56 (86.2%) | 43 (91.5%) | 36 (97.3%) | 0.0614 | 0.5521 | 0.0892 | 0.3781 |
| Tumor vascular contact | 0 | 0 | 0 | NA | NA | NA | NA |
| BMI ≥30 kg/m^2^ | 0 | 0 | 0 | NA | NA | NA | NA |
| Previous complex upper abdominal surgery | 0 | 0 | 0 | NA | NA | NA | NA |
|  |  |  |  |  |  |  |  |
| **ISGPS center classification**, n (%) |  |  |  |  |  |  |  |
| Grade A | 53 (81.5%) | 0 | 0 | **<0.0001** | **<0.0001** | **<0.0001** | NA |
| Grade B | 12 (18.5%) | 47 (100.0%) | 0 | 0.8495 | **<0.0001** | **0.0036** | **<0.0001** |
| Grade C | 0 | 0 | 37 (100.0%) | **<0.0001** | NA | **<0.0001** | **<0.0001** |
|  |  |  |  |  |  |  |  |
| **ISGPS surgeon classification**, n (%) |  |  |  |  |  |  |  |
| Grade A | 50 (76.9%) | 0 | 0 | **<0.0001** | **<0.0001** | **<0.0001** | NA |
| Grade B | 14 (21.5%) | 28 (59.6%) | 0 | 0.1675 | **<0.0001** | **0.0017** | **<0.0001** |
| Grade C | 1 (1.5%) | 19 (40.4%) | 37 (100.0%) | **<0.0001** | **<0.0001** | **<0.0001** | **<0.0001** |
|  |  |  |  |  |  |  |  |
| **ISGPS center and surgeon classification,** n (%) |  |  |  |  |  |  |  |
| Center A |  |  |  |  |  |  |  |
| Surgeon A | 38 (71.7%) | 0 | 0 | NA | NA | NA | NA |
| Surgeon B | 14 (26.4%) | 0 | 0 | NA | NA | NA | NA |
| Surgeon C | 1 (1.9%) | 0 | 0 | NA | NA | NA | NA |
|  |  |  |  |  |  |  |  |
| Center B |  |  |  |  |  |  |  |
| Surgeon A | 12 (100.0%) | 0 | 0 | **<0.0001** | **<0.0001** | NA | NA |
| Surgeon B | 0 | 28 (59.6%) | 0 | **0.0002** | **0.0002** | NA | NA |
| Surgeon C | 0 | 19 (40.4%) | 0 | **0.0059** | **0.0059** | NA | NA |
|  |  |  |  |  |  |  |  |
| Center C |  |  |  |  |  |  |  |
| Surgeon A | 0 | 0 | 0 | NA | NA | NA | NA |
| Surgeon B | 0 | 0 | 0 | NA | NA | NA | NA |
| Surgeon C | 0 | 0 | 37 (100.0%) | NA | NA | NA | NA |
|  |  |  |  |  |  |  |  |
| Age, median (IQR), years | 72 (63.5-78) | 72 (65-77) | 70 (64-76) | 0.6882 | 0.9412 | 0.4623 | 0.4168 |
| Male gender, n (%) | 40 (61.5%) | 26 (55.3%) | 19 (51.4%) | 0.3018 | 0.5091 | 0.3165 | 0.7174 |
| BMI, median (IQR), kg/m^2^ | 24 (22.1-25.4) | 25.3 (22.9-27.2) | 24.2 (22.1-26.2) | 0.1259 | 0.0479 | 0.8129 | 0.1558 |
| ASA score, n (%)  I  II  III  IV  >II | 1 (1.5%)  38 (58.5%)  25 (38.5%)  1 (1.5%)  26 (40.0%) | 5 (10.6%)  29 (61.7%)  11 (23.4%)  2 (4.3%)  13 (27.7%) | 0  7 (18.9%)  29 (78.4%)  1 (2.7%)  30 (81.1%) | 0.9474  **0.0005**  **0.0009**  0.6363  **0.0005** | 0.0807  0.7299  0.0922  0.5711  0.1761 | 1.0000  **0.0001**  **0.0001**  1.0000  **<0.0001** | 0.0638  **0.0001**  **<0.0001**  1.0000  **<0.0001** |
| Pre-operative oncology treatments, n (%)  Chemotherapy  Radiotherapy | 4 (6.2%)  1 (1.5%) | 2 (4.3%)  1 (2.1%) | 1 (2.7%)  0 | 0.4183  0.5813 | 1.0000  1.0000 | 0.6507  1.0000 | 1.0000  1.0000 |
| Approach, n (%)  Laparoscopy  Hybrid laparoscopy  All laparoscopic approaches  Robotic | 19 (29.2%)  3 (4.6%)  22 (33.9%)  43 (66.2%) | 17 (36.2%)  0  17 (36.2%)  30 (63.8%) | 0  0  0  37 (100.0%) | **0.0037**  0.0778  **0.0007**  **0.0007** | 0.4377  0.2628  0.7989  0.7989 | **<0.0001**  0.5518  **<0.0001**  **<0.0001** | **<0.0001**  NA  **<0.0001**  **<0.0001** |
| Operative time, mean$\pm$SD, min | 506$\pm$13.8 | 464$\pm$16.2 | 531$\pm$18.6 | **0.0230** | 0.0900 | 0.3212 | **0.0011** |
| Blood loss, median (IQR), mL | 200 (125-500) | 175 (100-300) | 120 (50-300) | 0.0113 | 0.1403 | **0.0045** | 0.0784 |
| Blood transfusion, median (IQR), mL | 0 (0-0) | 0 (0-0) | 0 (0-0) | **0.0339** | **0.0087** | 0.5566 | 0.0571 |
| Conversion, n (%)  Due to intraoperative complications  All reasons | 1 (1.5%)  10 (15.4%) | 1 (2.1%)  2 (4.3%) | 0  1 (3.2%) | 0.5813  **0.0284** | 1.0000  0.0704 | 1.0000  0.0977 | 1.0000  1.0000 |
| Firm pancreatic stump, n (%) | 22 (35.5%) | 22 (51.2%) | 17 (47.2%) | 0.1914 | 0.1093 | 0.2524 | NA |
| Pancreatic anastomosis, n (%)  Pancreatico-jejunostomy  Pancreatico-gastrostomy  Duct occlusion | 58 (90.6%)  4 (6.3%)  2 (3.1%) | 47 (100.0%)  0  0 | 37 (100.0%)  0  0 | **0.0112**  **0.0396**  0.1485 | 0.0379  0.1359  0.5073 | 0.0830  0.2936  0.5311 | NA  NA  NA |
| Length of hospital stay, median (IQR), days | 13 (10-22) | 16 (11-29) | 14 (9.5-19) | 0.2659 | 0.1351 | 0.9741 | 0.2181 |
| Postoperative complications, n (%)  None  Grade 1  Grade 2  Grade 3a  Grade 3b  Grade 4a  Grade 4b  Grade 5  > Grade 2 | 0  13 (20.0%)  21 (32.3%)  11 (16.9%)  1 (12.3%)  3 (4.6%)  0  9 (13.9%)  31 (47.7%) | 0  8 (17.0%)  24 (51.1%)  9 (19.2%)  3 (6.4%)  1 (2.1%)  0  2 (4.3%)  15 (31.9%) | 23 (62.2%)  3 (8.1%)  6 (16.2%)  4 (10.8%)  1 (2.7%)  0  0  0  8 (21.6%) | **<0.0001**  0.1289  0.2458  0.4910  0.0763  0.1573  NA  **0.0070**  **0.0066** | NA  0.6902  0.0457  0.7615  0.3529  0.6380  NA  0.1158  0.0939 | **<0.0001**  0.1584  0.0765  0.5633  0.1503  0.5518  NA  0.0245  **0.0092** | **<0.0001**  0.3324  **0.0009**  0.3707  0.6269  1.0000  NA  0.5011  0.3331 |
| Failure-to-rescue, n (%) | 9 (31.0%) | 2 (13.3%) | 0 | **0.0366** | 0.2816 | 0.1589 | 0.5257 |
| Post-operative pancreatic fistula, n (%)  Biochemical leak  Grade B/C  Grade C | 7 (10.8%)  16 (24.6%)  4 (6.2%) | 14 (29.8%)  7 (14.9%)  0 | 2 (5.4%)  3 (8.1%)  0 | 0.8487  **0.0297**  **0.0410** | **0.0143**  0.2088  0.1377 | 0.4816  0.0621  0.2937 | **0.0050**  0.5010  NA |
| Biliary leak, n (%) | 19 (15.4%) | 4 (8.5%) | 1 (2.7%) | **0.0367** | 0.3881 | 0.0533 | 0.3781 |
| Intestinal leak, n (%) | 2 (3.1%) | 3 (6.4%) | 0 | 0.5494 | 0.6480 | 0.5331 | 0.2517 |
| Chyle leak, n (%) | 2 (3.1%) | 4 (8.5%) | 0 | 0.6518 | 0.2358 | 0.5331 | 0.1267 |
| Delayed gastric emptying, n (%)  Grade B/C  All grades | 8 (12.3%)  10 (15.4%) | 8 (17%)  17 (36.2%) | 3 (8.1%)  3 (8.1%) | 0.6630  0.7298 | 0.4817  **0.0111** | 0.7419  0.3660 | 0.0324  **0.0039** |
| Post-pancreatectomy hemorrhage, n (%) | 17 (26.2%) | 8 (17.0%) | 1 (2.7%) | **0.0029** | 0.2520 | **0.0024** | 0.0708 |
| Abdominal fluid collections, n (%)  Drained abdominal fluid collection  All abdominal fluid collection | 12 (18.5%)  22 (33.9%) | 5 (10.6%)  12 (25.5%) | 4 (10.8%)  9 (24.3%) | 0.2362  0.2696 | 0.2548  0.3450 | 0.4014  0.3148 | 1.0000  0.8990 |
| Reoperation, n (%) | 14 (21.5%) | 5 (10.6%) | 1 (2.7%) | **0.0058** | 0.1293 | **0.0090** | 0.2222 |
| Tumor type*****, n (%)  Malignant tumor  PDAC | 57 (87.7%)  28 (43.1%) | 40 (87.0%)  23 (50.0%) | 23 (85.2%)  16 (59.3%) | 0.7531  0.1530 | 0.9084  0.4709 | 0.7423  0.1571 | 0.8317  0.4439 |
| Lymph nodes, median***** (IQR) Examined  Positive | 22 (17-32)  1 (0-3.8) | 20 (15-30)  1 (0-3.8) | 40 (33-50)  4.5 (2.8-8.3) | **<0.0001**  **0.0003** | 0.2153  0.6799 | **<0.0001**  **0.0002** | **<0.0001**  **0.0004** |

***** Data missing on 11 cases for tumor type, on 20 cases on number of examined lymph nodes, and on 35 cases for positive lymph nodes; PDAC: Pancreatic Ductal Adenocarcinoma; **Bold= significant p**

**Supplementary table 3**– Preoperative and postoperative results classified for the different ISGPS experience groups considering only ISGPS complexity grade C procedures

|  | **Experience Scoring** | | |  |  |  |  |
| --- | --- | --- | --- | --- | --- | --- | --- |
|  | **Grade A** | **Grade B** | **Grade C** | **p** | **pAB** | **pAC** | **pBC** |
| **ISGPS complexity parameters**, n (%) |  |  |  |  |  |  |  |
| Main pancreatic duct diameter ≤3 mm***** | 89 (89%) | 101 (94.4%) | 31 (67.4%) | **0.0050** | 0.1579 | **0.0015** | **<0.0001** |
| Common bile duct diameter ≤5 mm | 97 (97%) | 105 (98.1%) | 40 (83.3%) | **0.0030** | 0.6743 | **0.0055** | **0.0005** |
| Tumor vascular contact | 12 (12%) | 2 (1.9%) | 18 (37.5%) | **0.0013** | 0.0045 | **0.0003** | **<0.0001** |
| BMI ≥30 kg/m^2^ | 11 (11%) | 23 (21.5%) | 4 (8.3%) | 0.8576 | 0.0417 | 0.7745 | 0.0653 |
| Previous complex upper abdominal surgery | 0 (0%) | 0 (0%) | 1 (2.1%) | 0.1003 | NA | 0.3243 | 0.3097 |
|  |  |  |  |  |  |  |  |
| **ISGPS center classification**, n (%) |  |  |  |  |  |  |  |
| Grade A | 74 (74.0%) | 0 | 0 | **<0.0001** | **<0.0001** | **<0.0001** | NA |
| Grade B | 26 (26.0%) | 107 (100.0%) | 0 | 0.8481 | **<0.0001** | **<0.0001** | <0.0001 |
| Grade C | 0 | 0 | 48 (100.0%) | **<0.0001** | NA | **<0.0001** | <0.0001 |
|  |  |  |  |  |  |  |  |
| **ISGPS surgeon classification**, n (%) |  |  |  |  |  |  |  |
| Grade A | 90 (90.0%) | 0 | 0 | **<0.0001** | **<0.0001** | **<0.0001** | NA |
| Grade B | 8 (8.0%) | 72 (67.3%) | 0 | 0.1264 | **<0.0001** | 0.0541 | <0.0001 |
| Grade C | 2 (2.0%) | 35 (32.7%) | 48 (100.0%) | **<0.0001** | **<0.0001** | **<0.0001** | <0.0001 |
|  |  |  |  |  |  |  |  |
| **ISGPS center and surgeon classification,** n (%) |  |  |  |  |  |  |  |
| Center A |  |  |  |  |  |  |  |
| Surgeon A | 64 (86.5%) | 0 | 0 | NA | NA | NA | NA |
| Surgeon B | 8 (10.8%) | 0 | 0 | NA | NA | NA | NA |
| Surgeon C | 2 (2.7%) | 0 | 0 | NA | NA | NA | NA |
|  |  |  |  |  |  |  |  |
| Center B |  |  |  |  |  |  |  |
| Surgeon A | 26 (100.0%) | 0 | 0 | **<0.0001** | **<0.0001** | NA | NA |
| Surgeon B | 0 | 72 (67.3%) | 0 | **<0.0001** | **<0.0001** | NA | NA |
| Surgeon C | 0 | 35 (32.7%) | 0 | **0.0003** | **0.0003** | NA | NA |
|  |  |  |  |  |  |  |  |
| Center C |  |  |  |  |  |  |  |
| Surgeon A | 0 | 0 | 0 | NA | NA | NA | NA |
| Surgeon B | 0 | 0 | 0 | NA | NA | NA | NA |
| Surgeon C | 0 | 0 | 48 (100.0%) | NA | NA | NA | NA |
|  |  |  |  |  |  |  |  |
| Age, median (IQR), years | 67 (56.3-74) | 69 (60-76) | 65.5 (56.3-71.8) | 0.1132 | 0.1858 | 0.2694 | 0.0560 |
| Male gender, n (%) | 48 (48.0%) | 61 (57.0%) | 29 (60.4%) | 0.1176 | 0.1945 | 0.1652 | 0.6910 |
| BMI, median (IQR), kg/m^2^ | 24.1 (22-26.6) | 25.2 (23.2-28.7) | 24.4 (22.6-27.7) | **0.0265** | 0.0092 | 0.5108 | 0.1170 |
| ASA score, n (%)  I  II  III  IV  >II | 8 (8.0%)  54 (54.0%)  37 (37.0%)  1 (1.0%)  38 (38.0%) | 7 (6.5%)  59 (55.0%)  41 (38.3%)  0  41 (38.3%) | 1 (2.1%)  15 (31.3%)  32 (66.7%)  0  32 (66.7%) | 0.1886  **0.0278**  **0.0027**  0.2772  **0.0042** | 0.6860  0.8692  0.8450  0.4831  0.9625 | 0.1586  **0.0094**  **0.0007**  1.0000  **0.0015** | 0.4362  **0.0087**  **0.0016**  NA  **0.0011** |
| Pre-operative oncology treatments, n (%)  Chemotherapy  Radiotherapy | 8 (8%)  0 (0%) | 2 (1.9%)  1 (0.9%) | 5 (10.4%)  0 (0%) | 0.9830  0.7807 | 0.0523  1.0000 | 0.7574  NA | 0.0299  1.0000 |
| Approach, n (%)  Laparoscopy  Hybrid laparoscopy  All laparoscopic approaches  Robotic | 20 (20%)  11 (11%)  31 (31%)  69 (69%) | 48 (44.8%)  8 (7.5%)  56 (52.3%)  51 (47.7%) | 0 (0%)  0 (0%)  0 (0%)  48 (100%) | 0.2367  **0.0206**  **0.0170**  **0.0170** | **0.0001**  0.3803  **0.0019**  **0.0019** | **0.0002**  **0.0166**  **<0.0001**  **<0.0001** | **<0.0001**  0.0587  **<0.0001**  **<0.0001** |
| Operative time, mean$\pm$SD, min | 518.9$\pm$10.5 | 482.9$\pm$10.2 | 559.6$\pm$15.2 | **<0.0001** | 0.0198 | 0.0314 | **<0.0001** |
| Blood loss, median (IQR), mL | 200 (100-300) | 200 (150-350) | 150 (100-245) | **0.0205** | 0.0535 | 0.2592 | **0.0107** |
| Blood transfusion, median (IQR), mL | 0 (0-0) | 0 (0-0) | 0 (0-0) | 0.0615 | 0.0183 | 0.5053 | 0.1742 |
| Conversion, n (%)  Due to intraoperative complications  All reasons | 3 (3%)  16 (16.2%) | 2 (1.9%)  6 (5.6%) | 0  0 | 0.2230  **0.0005** | 0.6743  **0.0143** | 0.5512  **0.0029** | 1.0000  0.1801 |
| Firm pancreatic stump, n (%) | 17 (17.4%) | 17 (16.2%) | 11 (23.9%) | 0.4441 | 0.8255 | 0.3533 |  |
| Pancreatic anastomosis, n (%)  Pancreatico-jejunostomy  Pancreatico-gastrostomy  Duct occlusion | 85 (85.0%)  12 (12.0%)  3 (3.0%) | 107 (100.0%)  0  0 | 44 (95.7%)  2 (4.4%)  0 | **0.0012**  **0.0082**  0.0601 | **<0.0001**  **0.0001**  0.1110 | 0.0934  0.2263  0.5518 | 0.0890  0.0890  NA |
| Length of hospital stay, median (IQR), days | 17 (11-28) | 18 (9-32) | 20.5 (13.3-30.8) | 0.3912 | 0.7019 | 0.1426 | 0.3330 |
| Postoperative complications, n (%)  None  Grade 1  Grade 2  Grade 3a  Grade 3b  Grade 4a  Grade 4b  Grade 5  > Grade 2 | 0  18 (18.0%)  29 (29.0%)  24 (24.0%)  16 (16.0%)  5 (5.0%)  3 (3.0%)  5 (5.0%)  5 (53%) | 0  12 (11.2%)  38 (35.5%)  25 (23.4%)  6 (5.6%)  10 (9.4%)  4 (3.7%)  12 (11.2%)  57 (53.3%) | 21 (43.8%)  2 (4.2%)  15 (31.3%)  5 (10.4%)  2 (4.2%)  1 (2.1%)  0  2 (4.2%)  14 (29.2%) | **<0.0001**  **0.0147**  0.6191  0.0953  **0.0078**  0.7954  0.4116  0.7763  **0.0192** | NA  0.1658  0.3168  0.9144  **0.0153**  0.2879  1.0000  0.1308  0.9689 | **<0.0001**  0.0214  0.8483  0.0753  0.0574  0.6642  0.5512  1.0000  **0.0064** | **<0.0001**  0.2283  0.6048  0.0781  0.7078  0.1742  0.3115  0.2283  **0.0054** |
| Failure-to-rescue, n (%) | 5 (9.4%) | 12 (21.1%) | 2 (14.3%) | 0.2641 | 0.1163 | 0.6304 | 0.5685 |
| Post-operative pancreatic fistula, n (%)  Biochemical leak  Grade B/C  Grade C | 21 (21.0%)  35 (35.0%)  11 (11.0%) | 26 (24.3%)  34 (31.8%)  7 (6.6%) | 2 (4.2%)  10 (20.8%)  1 (2.1%) | 0.0511  0.1010  **0.0466** | 0.5713  0.6229  0.2553 | **0.0073**  0.0795  0.1041 | **0.0027**  0.1624  0.4362 |
| Biliary leak, n (%) | 14 (14.0%) | 9 (8.4%) | 2 (4.2%) | **0.0477** | 0.2011 | 0.0915 | 0.5045 |
| Intestinal leak, n (%) | 2 (2.0%) | 8 (7.5%) | 0 | 0.9862 | 0.1029 | 1.0000 | 0.0587 |
| Chyle leak, n (%) | 10 (10.0%) | 4 (3.7%) | 3 (6.3%) | 0.2269 | 0.0971 | 0.5486 | 0.6774 |
| Delayed gastric emptying, n (%)  Grade B/C  All grades | 16 (16.0%)  25 (25.0%) | 24 (22.4%)  32 (29.9%) | 8 (16.7%)  11 (22.9%) | 0.6963  0.9795 | 0.2417  0.4297 | 0.9180  0.7821 | 0.5213  0.3688 |
| Post-pancreatectomy hemorrhage, n (%) | 1 (23.0%) | 28 (26.2%) | 3 (6.3%) | 0.0605 | 0.5971 | **0.0115** | **0.0042** |
| Abdominal fluid collections, n (%)  Drained abdominal fluid collection  All abdominal fluid collection | 31 (31.0%)  45 (45.0%) | 21 (19.6%)  39 (36.5%) | 10 (20.8%)  18 (37.5%) | 0.0965  0.2803 | 0.0594  0.2106 | 0.1958  0.3877 | 0.8621  0.9001 |
| Reoperation, n (%) | 22 (22.0%) | 17 (15.9%) | 4 (8.3%) | **0.0354** | 0.2611 | 0.0408 | 0.3097 |
| Tumor type, n (%)  Malignant tumor*  PDAC* | 86 (86.0%)  38 (38.0%) | 97 (90.7%)  30 (28.0%) | 32 (91.4%)  16 (45.7%) | 0.2722  0.9133 | 0.2959  0.1272 | 0.5582  0.4227 | 1.0000  0.0524 |
| Lymph nodes, median***** (IQR) Examined  Positive | 24 (17.5-34)  1 (0-4) | 20 (16-26)  1 (0-2) | 37.5 (28-49)  2.5 (0-5.8) | **<0.0001**  **0.0100** | **0.0039**  0.1412 | **<0.0001**  **<0.0001** | **<0.0001**  **0.0025** |

***** Data missing on 2 cases for main pancreatic duct size, on 13 cases for tumor type, on 22 cases on number of examined lymph nodes, and on 50 cases for positive lymph nodes; PDAC: Pancreatic Ductal Adenocarcinoma; **Bold= significant p.**

**Supplementary figure 1.** Study flowchart

**
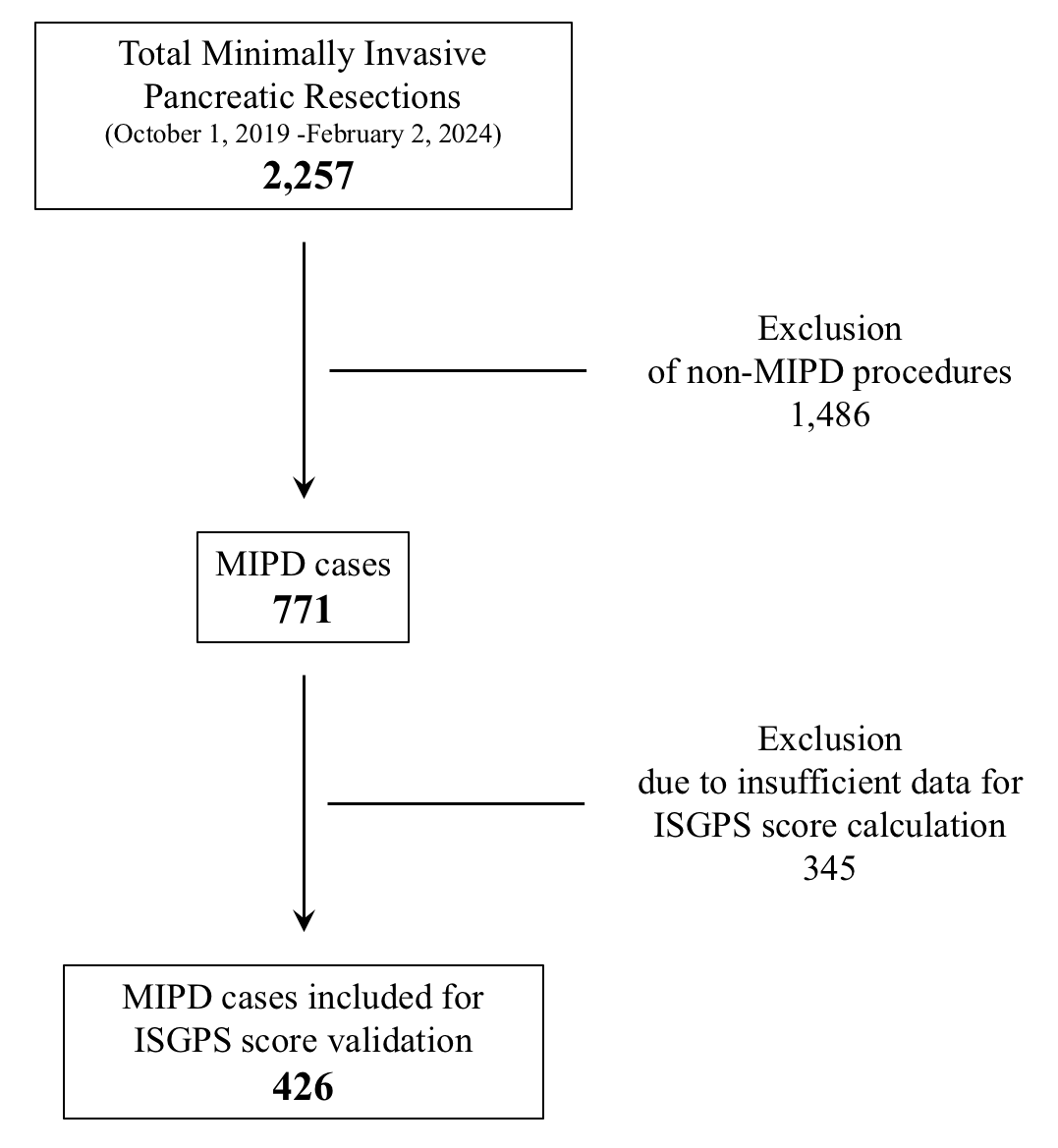
**
